# Epidemiological Insights into Small Ruminant Lentiviruses in Portuguese Production Systems

**DOI:** 10.3390/ani16081251

**Published:** 2026-04-18

**Authors:** João Jacob-Ferreira, Ana Cláudia Coelho, Ana Grau Vila, Delia Lacasta, Ramiro Valentim, Hélder Quintas

**Affiliations:** 1 Centro de Investigação de Montanha (CIMO), Laboratório Associado Para a Sustentabilidade e Tecnologia nas Regiões de Montanha (SusTEC), Campus de Santa Apolónia, 5300-253 Bragança, Portugal; valentim@ipb.pt (R.V.); helder5tas@ipb.pt (H.Q.); 2 Animal and Veterinary Research Centre, Associate Laboratory for Animal and Veterinary Sciences (AL4AnimalS), 5000-801 Vila Real, Portugal; accoelho@utad.pt; 3 Servicio de Sanidad Animal, Dirección General de Producción Agropecuaria e Infraestructuras Agrarias, Consejería de Agricultura y Ganadería, Junta de Castilla y León, 47014 Valladolid, Spain; gravilan@jcyl.es; 4 Departamento de Patología Animal, Instituto Agroalimentario de Aragón-IA2, Universidad de Zaragoza-CITA, 50013 Zaragoza, Spain; delialacasta@gmail.com

**Keywords:** small ruminant lentiviruses, sheep, goat, seroprevalence, risk factors

## Abstract

Small ruminant lentiviruses are longstanding viral infections affecting sheep and goats worldwide, resulting in reduced efficiency and economic losses. In Portugal, updated epidemiological data are scarce. The aim of this study was to assess the seroprevalence and risk factors for SRLV in Portugal. The study was conducted in Portuguese flocks of ovine and caprine species. Flocks were randomly chosen, and producers were invited to answer a questionnaire. The indirect ELISA test, ID Screen^®^ MVV/CAEV Indirect, was made to detect infection. We collected samples from 59 flocks, of which 55.93% (CI 95%: 43.26–68.60%) had at least one positive animal. Of these flocks, 1302 individual samples presented a seroprevalence of 32.95% (CI 95%: 30.08–35.81%). Regarding the risk factor analysis, the multivariable mixed-effects logistic regression model at the individual level identified variables with increased odds of SRLV seropositivity. Caprine species (OR = 2.47; CI 95%: 1.01–6.03), non-autochthonous breed (OR = 2.95; CI 95%: 1.23–7.06), animals older than two years old (OR = 1.95; CI 95%: 1.29–2.94), dairy aptitude (OR = 8.15; CI 95%: 2.53–26.24), unknown serostatus of newly acquired animals (OR = 9.41; CI 95%: 2.93–30.23) and participation in livestock competitions (OR = 4.25; CI 95%: 1.42–12.73) were significantly associated with increased odds of seropositivity. SRLV has been confirmed in both regions of Portugal studied, with a significant regional disparity that is likely attributable to differences in management practices. The identification of risk factors specific to each production system is crucial for the development and implementation of voluntary control programs.

## Simple Summary

Ovine and caprine species may be affected by Small Ruminant Lentiviruses, causing a chronic, progressive, and long-standing disease. For establishing a diagnosis, laboratory methods are essential. In Portugal, the highly diverse production systems across different regions contribute to the high profitability and social value of small ruminant production. The aim of this study was to assess the seroprevalence and risk factors for SRLV in Portugal. Almost 56% of flocks had at least one seropositive animal to SRLV, and nearly one-third of the animals were positive to SRLV. The foremost variables associated with SRLV seropositivity were caprine species, non-autochthonous breeds, animals older than 2 years, dairy flocks, unknown serological status of newly acquired animals and participation in livestock competitions. We observed an elevated seroprevalence of SRLV in Portuguese flocks of small ruminants, probably associated with the production system. The identified risk factors reinforce the need to improve biosafety measures, promote regular tracking, and implement structured and voluntary control programs to reduce virus dissemination and ensure the profitability of small ruminant flocks in Portugal.

## 1. Introduction

Sheep and goats are affected by a wide range of diseases that impact animal health and welfare, with consequent repercussions on flock profitability. Among these, wasting diseases—particularly those caused by Small Ruminant Lentiviruses (SRLV)—are of major importance, as they are widely distributed worldwide [1]. Lentivirus infection is persistent and leads to a chronic, insidious, and progressive disease that may affect the lungs, central nervous system, mammary gland, and joints [2,3,4,5,6]. In this setting, the health and welfare of infected animals are severely compromised, resulting in substantial economic losses that are difficult to quantify accurately [7]. Premature culling is common, primarily due to suboptimal productive performance [8,9]. This is particularly relevant in dairy systems, where SRLV infection may reduce both milk yield and quality [10,11,12,13], although findings are not entirely consistent across the literature. This disease lacks an effective treatment or vaccine for prevention, despite efforts by many research groups [14].

Taxonomically, SRLV belongs to the family Retroviridae and the genus Lentivirus and is classified into five distinct genotypes (A-E), some of which comprise multiple subtypes [15,16]. The characterization of this group of phylogenetically related viruses has led to the recognition that apparently distinct diseases, such as Maedi-Visna and Caprine Arthritis–Encephalitis (CAE), share a common etiological basis [3]. This is further supported by the ability of certain subtypes to infect and be transmitted between sheep and goats [17,18].

The distribution of SRLV prevalence is highly heterogeneous worldwide, with marked variations observed between continents and even among different regions within the same continent [1,19,20,21,22,23,24]. Nevertheless, high individual and flock-level prevalences have been consistently reported in several European countries, particularly in association with high small ruminant population density and intensive management systems [1,25]. Similarly, in goats, studies report relatively high prevalence rates of SRLV infection, strongly influenced by factors such as geographic region and production system [26,27,28].

SRLV transmission may occur through multiple routes. Vertically, infected progenitors can transmit the virus to their offspring by ingesting infected colostrum and milk. Although many authors do not consider this route to be the primary form of transmission [29,30,31]. Horizontally, infection may occur via inhalation of viral particles from infected animals [32]. This route is considered the principal mode of transmission in intensive production systems or under housing conditions involving prolonged confinement [33,34]. Intrauterine transmission, seminal transmission, indirect transmission via fomites and other potential transmission routes have been described in the literature, although they appear to play a less significant role [35,36,37]. However, it remains unclear whether these routes consistently result in real infection in either the progenitor or the offspring [38].

The diagnosis of SRLV infection is based on laboratory testing, including serological and molecular assays. Currently, the most widely used diagnostic methods are enzyme-linked immunosorbent assay (ELISA) and polymerase chain reaction (PCR) or reverse transcription (RT-PCR) [39,40]. Clinical and pathological evaluation may not be reliable for timely and accurate diagnosis, as most infected animals remain asymptomatic or develop characteristic lesions only at an advanced stage of the disease [41,42,43]. Delayed seroconversion, seroreversion, genetic heterogeneity of viral strains and low viral load are among the factors that complicate laboratory detection [12,44,45]. In the absence of effective detection strategies, implementing control programs becomes challenging. Many control programs worldwide have adopted multiple diagnostic tests, combined with enhanced biosecurity measures at the flock level, to improve disease control and limit viral spread [46,47,48].

In Portugal, small ruminant farming is a long-standing traditional activity, predominantly oriented toward meat, milk, and wool production. Different production systems coexist, partly reflecting regional edaphoclimatic conditions, along with a wide diversity of breeds (both autochthonous and non-autochthonous). This distinction is particularly evident between regions up to the Tejo River, characterised by predominantly mountainous terrain, and regions to the south of the Tejo River, where plains predominate. Nowadays, the establishment of more intensive and industrialized farming operations has also been observed, distributed across the country, particularly with a dairy-oriented production focus. Human activities are recognized as influential factors in disease ecology, including SRLV epidemiology [16]. Understanding how specific risk factors influence disease dynamics is essential for developing tailored, more effective control programs [45].

Accordingly, the primary objective of this study was to investigate the seroepidemiology of SRLV and identify associated variables across different small ruminant production systems in Portugal.

## 2. Materials and Methods

### 2.1. Study Area and Animal Populations

Portugal is the westernmost country in continental Europe, occupying a geographically strategic position. Overall, it is characterised by a Mediterranean climate, with a cold, wet winter and a hot, dry summer. However, several regional climatic mosaics can be identified, resulting from the interaction between Atlantic and Mediterranean influences, as well as from the country’s diverse orography and topography [49]. The Northern and Central regions (north of the Tejo River) are predominantly mountainous, whereas the Southern region (south of the Tejo River) is characterised by more extensive lowland areas. For this reason, the study design considered a division of the country into Northern/Central and Southern regions in order to account for these marked geographic and production-system differences.

According to the Portuguese Institute for Financing Agriculture and Fisheries (IFAP, I.P. Lisboa, Portugal), Portugal has approximately 2,170,000 sheep and 250,000 goats, primarily raised for meat and milk production. There is considerable breed diversity across the country, including both autochthonous and exotic breeds, as well as various crossbreeds. Three main small ruminant production systems can be pointed out as the most representative. Extensive production systems are predominantly associated with the vast lowland areas. Traditional pastoralism or semi-extensive production systems are particularly characteristic of hill and mountainous regions. Additionally, intensive production systems are considered typical of more specialized and highly managed production contexts [50].

### 2.2. Study Design and Sampling

A cross-sectional study was conducted using a two-stage (cluster) sampling design, in which small ruminant flocks were randomly selected from different regions across the country based on the official animal health database (PISA.net, Digidelta Software (https://www.digidelta-software.com/), Leiria Portugal). Partnerships were established with the veterinarians responsible for the selected flocks, and farmers were invited to enrol in the study on a voluntary basis. Only flocks comprising a minimum of 20 animals were included.

The number of animals to be sampled was estimated using the formula *n* = (1.96)^2^*p*(1 − *p*)/*d*^2^ [51]. This sample size provides 95% confidence at an expected prevalence of 15%. Although this calculation assumes simple random sampling, it was used as a pragmatic approximation to guide field sampling, acknowledging that the clustered nature of the data may introduce a design effect that was not explicitly accounted for. However, clustering at the flock level was explicitly addressed in the analytical phase through the use of mixed-effects models, thereby accounting for intra-cluster correlation. This sample size also allows detection of a minimum within-flock prevalence of approximately 1%, balancing statistical precision and operational feasibility.

Due to logistical and economic constraints, it was not feasible to sample all animals within each flock. Therefore, a random intra-flock sampling strategy was implemented, selecting between 14 and 19 animals per flock using random numbers generated from a complete list of animals. This approach reflects the hierarchical structure of the data, with animals nested within flocks.

Within each flock, animals were randomly selected using random numbers generated from a complete list of animals. Blood samples were collected from sheep and goats aged at least six months during routine flock health visits conducted by the veterinarians responsible for each flock.

Sampling procedures and laboratory testing were carried out between May 2022 and December 2024. A flock was defined as seropositive for SRLV if at least one seropositive animal was identified. This definition was used exclusively for descriptive flock-level analysis and not for modelling risk factors. Risk factors and health management protocols were recorded in all small ruminant flocks using a structured questionnaire. The questionnaire was developed by the authors and had been applied in a previous study [52]. It comprised 40 closed-ended questions with predefined response options.

### 2.3. Serological Analysis

Blood samples (10 mL) were collected from each animal by jugular venipuncture into 10 mL tubes (Vacutainer^®^, Becton Dickinson, Plymouth, UK) containing a clot activator. The blood samples were allowed to clot at room temperature. Serum was subsequently separated by centrifugation at 200× *g* for 10 min and stored at −20 °C until analysis.

Serological analysis of the samples was performed at the Laboratorio Provincial de Sanidad Animal de Zamora. SRLV infection status was determined using a commercial indirect ELISA test (ID Screen^®^ MVV/CAEV Indirect, Innovative Diagnostics, Grabels, France), following the manufacturer’s instructions. This ELISA test uses a mixture of peptide antigens, thereby improving test performance. It provides a clear distinction between positive and negative results, with high sensitivity and the ability to detect all genotypes (including A, B and E) with high specificity. According to the manufacturer’s data, at a 95% confidence interval (CI), the test’s diagnostic sensitivity and specificity are approximately 91.7% and 98.9%, respectively [53].

### 2.4. Epidemiological and Statistical Analysis

The epidemiological questionnaires collected in the field were entered into Microsoft^®^ Excel^®^ for Microsoft 365 MSO (version 2601, build 16.0.19628.20166). The SRLV infection status of each sample was matched to the corresponding responses obtained from the questionnaire of the flock of origin. The calculation of confidence intervals for apparent seroprevalence and the calculation of true seroprevalence were performed using WinEpi 2.0 Software [54]. Statistical analyses were performed using JMP^®^ Statistical Discovery software, version 19.

Descriptive statistics were initially conducted to summarise seroprevalence data at both the flock and individual levels. Associations between SRLV seropositivity and categorical variables were initially explored using Pearson’s chi-square (χ^2^) test.

Given the hierarchical structure of the data, with animals nested within flocks, risk factor analysis was performed at the individual level using mixed-effects logistic regression models, including flock as a random effect to account for intra-cluster correlation. Univariable mixed-effects logistic regression analysis was first used to evaluate potential associations between each explanatory variable and SRLV seropositivity. Variables showing statistical significance in the univariable analysis (*p* < 0.05) and considered biologically plausible were included in the preliminary multivariable model.

Prior to model fitting, collinearity among candidate explanatory variables was assessed using variance inflation factors (VIF) and examination of correlation matrices. Variables showing evidence of high collinearity were not included simultaneously in the multivariable model. Variables were then sequentially removed according to statistical significance and model stability. Variable selection was based on statistical and epidemiological considerations, including biological plausibility and results from univariable screening. Potential explanatory variables were initially screened and subsequently included in the multivariable model based on predefined criteria. Confounding was assessed by examining changes in the estimated OR of the remaining variables within the mixed-effects framework; variables causing a change of 10% or more in at least one OR were considered confounders and would be retained regardless of statistical significance. However, no variables met this criterion, and therefore none were retained in the final model as confounders.

Model fit was evaluated using likelihood ratio tests and inspection of the variance component associated with the random effect. Model stability was assessed by examining parameter estimates, standard errors, and model convergence diagnostics. Sensitivity analyses were performed by comparing alternative model specifications and by sequentially removing variables to evaluate the robustness of the final model. Statistical significance was defined as *p* < 0.05.

## 3. Results

### 3.1. Seroprevalence

Samples from 59 Portuguese small ruminant flocks were analysed, with Table 1 showing SRLV seroprevalence in the flocks studied.

A total of 33 out of 59 flocks had at least one SRLV-seropositive animal, resulting in an apparent prevalence of 55.93% (CI 95%: 43.26–68.60%). Considering the test’s diagnostic (91.7%) and specificity (98.9%), the estimated true national prevalence was 60.52% (CI 95%: 48.05–72.99%). At the regional level, the apparent prevalence in the Northern and Central regions was 82.14% (CI 95%: 67.96–96.33%), whereas the Southern region showed a lower prevalence of 32.26% (CI 95%: 15.80–48.71%). Analysis of the previous table indicated that, at the national level, SRLV prevalence was markedly higher in goat herds than in sheep or mixed flocks. This pattern differed in the Northern and Central regions, where sheep flocks exhibited a higher SRLV prevalence than goat herds.

Table 2 shows individual-level SRLV seroprevalence in the sheep and goats sampled in the study flocks.

An average of 17 blood samples per flock (17.49 ± 1.61) were collected. Among positive flocks, the mean proportion of seropositive animals was 58% (57.46 ± 30.80). The within-flock seroprevalence showed the following distribution: 26 flocks (44.10%) had no seropositive animals; 4 (6.78%) had fewer than 10% positive animals; 9 (15.25%) had between 10% and 50% positive animals; 16 (27.12%) had between 50% and 90% positive animals; and 4 (6.78%) had more than 90% positive animals. A total of 1.032 individual samples were collected, of which 340 tested positives in the diagnostic assay. The apparent prevalence was therefore 32.95% (CI 95%: 30.08–35.81%), and the estimated true prevalence was 35.15% (CI 95%: 32.24–38.06%). By species, 124 sheep (19.02%) and 216 goats (56.84%) were seropositive. Individual-level data highlights the markedly high SRLV prevalence in goats, both nationally and in the two studied regions. Notably, a very low infection prevalence was observed in sheep from the Southern region.

### 3.2. Risk Factors

Several factors that may influence the risk of SRLV transmission were assessed through a questionnaire answered by small ruminant producers. Associations between SRLV seropositivity and questionnaire responses are presented in Table 3.

Univariable analysis identified a highly statistically significant association (*p* < 0.001) between seropositivity and goat species, non-autochthonous breed, location in the Northern/Central region, non-extensive production system, dairy production purpose, flock size greater than 100 animals, permanent housing, producer age under 65 years old, absence of formal training in livestock production, prior knowledge of the disease, availability of regular veterinary assistance, implementation of artificial milk feeding management, and isolation of lambs at birth. A statistically significant association was also identified between seropositivity and older animals (age > 2 years old), non-commercial (hobby/secondary-type) producers, lack of knowledge regarding the SRLVs’ serological status of newly acquired animals, and participation in livestock competitions.

No statistically significant association (*p* > 0.05) was observed between seropositivity and animal sex, contact between sheep and goats, or the segregation of clinically ill animals.

Table 4 shows risk factors associated with SRLV seropositivity based on mixed-effects logistic regression analysis (flock included as random effect).

In the mixed-effects logistic regression analysis, fitted at the individual level with flock included as a random effect, several variables were associated with increased odds of SRLV seropositivity. In the univariable analysis, significant associations were observed for goat species, non-autochthonous breed, age older than 2 years, Northern/Central region, dairy production, non-extensive production system, and permanent confinement. In the final multivariable mixed-effects model, caprine species (OR = 2.47; 95% CI: 1.01–6.03; *p* = 0.047), non-autochthonous breed (OR = 2.95; 95% CI: 1.23–7.06; *p* = 0.015), age older than 2 years (OR = 1.95; 95% CI: 1.29–2.94; *p* = 0.002), dairy production aptitude (OR = 8.15; 95% CI: 2.53–26.24; *p* < 0.001), unknown serological status of newly acquired animals (OR = 9.41; 95% CI: 2.93–30.23; *p* < 0.001), and participation in competitions/markets (OR = 4.25; 95% CI: 1.42–12.73; *p* = 0.010) remained independently associated with SRLV seropositivity. Non-extensive production system showed a borderline association in the final model (OR = 2.87; 95% CI: 0.97–8.48; *p* = 0.056).

## 4. Discussion

The seroprevalence data from the surveyed flocks indicate that lentiviral infection is widespread across the studied regions of Portugal. At the national level, SRLV infection affected approximately 56% of the participating flocks and 33% of the sampled animals. In sheep, more than 42% of flocks were seropositive, with an individual seroprevalence of 19%. It is important to note that seroprevalence differed markedly between the two regions studied. In the North and Centre regions, 91% of flocks were SRLV-seropositive, with approximately 40% of individual samples testing positive. These findings are highly consistent with those previously reported in the northeastern area of the country [52]. Similar prevalence levels in sheep have also been described in certain regions of Spain [1,55,56,57] and in other European countries [25]. Conversely, lower prevalence rates have been reported in other European countries and regions [34,58], more closely resembling the seroprevalence observed in the Southern region in the present study. In the South, 19% of sheep flocks were seropositive, and only approximately 7% of individual animals tested positive.

Regarding the seroprevalence results obtained in this study for goats, it is noteworthy that both at the flock level (75%) and at the individual animal level (57%), the values were considerably higher than those observed in sheep. In the North and Centre regions, the flock-level seroprevalence in goats (71%) was slightly lower than that observed in sheep; however, at the individual level, seroprevalence was higher, with approximately 53% of goats testing positive. In the Southern region, the pattern differed from that described for sheep. In this region, 83% of goat herds had at least one SRLV-seropositive animal, and 64% of the sampled animals were seropositive. High flock-level seroprevalence values like those reported here have been described in Spain [26]. However, the individual-level seroprevalence observed in the present study exceeded that reported in the consulted literature [27,28,59,60].

It should be noted that variability in prevalence estimates across published studies may be attributable to differences in diagnostic methodologies [61]. Determining the true infection status of SRLV remains challenging due to the lack of a gold-standard test for the disease detection. Even when comparing results from ELISA-based assays, substantial variability may occur, as different commercial kits exhibit distinct sensitivities and specificities. These methodological differences must be considered when interpreting and comparing prevalence data across studies [40]. In Portugal, there is currently no official mandatory or voluntary national control programme for SRLV. Nevertheless, some producers, in collaboration with their veterinarians, have implemented sporadic control measures to mitigate the disease’s impact, particularly in intensive production systems.

Traditionally, small ruminant farming systems in Portugal differ across regions due to the long-standing adaptation of both humans and animals to local geographic and climatic conditions. In the North and Centre regions, the mountainous terrain and colder climate have historically required the confinement of sheep and goats in shelters during the night and on colder days. In contrast, the Southern region, characterised by extensive flat landscapes and a milder climate, has enabled small ruminants to be reared predominantly under grazing conditions, in many cases throughout the year, without the need for, or with minimal, confinement. More recently, intensive production systems have been established across different parts of the country, particularly for dairy production. These operations aim to professionalise the sector and enhance economic efficiency by maximising productivity and profitability.

In the present study, we aimed to assess, using statistical significance testing, the association between SRLV seropositivity and selected herd- and animal-level characteristics and management practices. We initially performed a univariable statistical analysis, which revealed statistically significant associations between seropositivity and the following factors: goat species, non-autochthonous breed, animals older than two years old, location in the North and Centre regions, non-extensive production systems, dairy-oriented herds, herd size greater than 100 animals, permanent confinement, non-professional producer status, absence of veterinary assistance, unknow serostatus of newly acquired animals and participation in livestock competitions. Other variables did not show statistically significant associations.

Considering the initial identification of significant associations in the univariable analysis, a multivariable modelling approach that accounted for the data structure was subsequently adopted. Specifically, given that animals were clustered within flocks, risk factor evaluation was conducted at the individual level using mixed-effects logistic regression, with flock as a random effect to control for within-flock dependency and potential clustering. The revised mixed-effects analysis strengthens the interpretation of the risk factor results by accounting for the hierarchical structure of the data and the lack of independence among animals sampled within the same flock. After adjustment for flock-level clustering, caprine species, non-autochthonous breed, older age, dairy production aptitude, introduction of animals with unknown serological status, and participation in livestock competitions or markets, remained associated with increased odds of SRLV seropositivity. These results support the view that host characteristics, production purpose, and management-related biosecurity practices play an important role in SRLV epidemiology in Portugal [52]. The association observed for non-extensive production systems should, however, be interpreted with caution, as it showed only borderline statistical support in the mixed-effects multivariable model. More broadly, because this was a cross-sectional study, the identified associations should not be interpreted as causal relationships.

Species was significantly associated with SRLV seropositivity. According to the model, goats were approximately 2.5 times more likely to be seropositive than sheep. The circulating genotypes within the country are currently under investigation by the authors, as this information is essential to better understand whether a species-related predisposition may be driven by viral factors influencing transmission dynamics. Several studies have identified co-rearing of sheep and goats within the same flock as a risk factor for SRLV transmission, as many viral subtypes can infect both species [57,62,63]. It is therefore crucial to determine whether the higher infection probability observed in goats is related to specific viral characteristics of subtypes that preferentially affect this species [18] or to inherent species-specific traits, such as their typically more interactive social behaviour within the herd, which may facilitate transmission.

Regarding breed, our study categorised animals as either autochthonous or non-autochthonous. Portugal is home to numerous indigenous breeds of sheep and goats, many of which have been gradually replaced by more productive exotic breeds. Our results demonstrated that non-autochthonous breeds had an almost threefold higher probability of SRLV seropositivity compared with autochthonous breeds. An association between specific breeds and SRLV infections has also been reported by several authors [18,45,55]. Susceptibility or resistance to SRLV may be influenced by the substantial genetic diversity observed among small ruminants, reflecting the wide range of breeds distributed worldwide [64,65,66].

With respect to the age of the sampled animals, only sheep and goats older than 6 months were included in the study. Animals were subsequently categorised into two age groups: older than 2 years and younger than 2 years. Animals older than 2 years were significantly associated with seropositivity, supporting the concept of often delayed seroconversion and highlighting the importance of transmission among adult animals within the flock [31]. This finding is consistent with previous reports in the literature [24,45,67,68].

Animals from dairy-oriented flocks exhibited more than an eightfold higher likelihood of infection compared to animals from meat-oriented flocks. In general, dairy flocks are characterised by higher animal density, longer periods of confinement, and increased production pressure, which may facilitate viral transmission and thereby explain this association [25].

Concerning the introduction of new stock, particularly replacement females and breeding males, producers were asked whether they purchased animals with prior knowledge of their SRLV serological status. A large proportion of producers reported acquiring animals without knowing their serological status. This practice was associated with a more than ninefold increase in the likelihood of disease occurrence compared with producers who purchased animals confirmed as seronegative. In the context of infectious diseases, the introduction of animals with positive or unknown serological status represents a well-recognised risk factor that producers should carefully consider [56,57,69].

Participation in livestock competitions or trading markets was also evaluated in our study. Animals from flocks that usually participate in these activities had a fourfold higher likelihood of infection compared to those that did not participate. Participation in competitions or trademark fairs gathers animals from different origins and encourages people to observe [69]. This way, these events predispose animals to infection by certain infectious agents due to increased contact. In many of these activities, it is only mandatory to provide proof of disease-free status from the official eradication programme (e.g., Brucellosis). So, biosafety measures are essential and may not be adequately followed by the organising committee of these events.

The production system exhibited a borderline significant association with SRLV seropositivity; animals in non-extensive systems were approximately three times more likely to be infected than those in extensive systems. This finding aligns with previous [25,45,55,70,71,72] suggesting that higher stocking densities, prolonged confinement, and increased production pressure typical of intensive systems predispose animals to infection. However, as this association did not reach significance in the final multivariable model, these results should be interpreted with caution.

These findings underscore the need to develop and implement effective voluntary SRLV control programs tailored to the specific seroprevalence within each flock. In flocks with high prevalence-where the financial burden of implementing comprehensive eradication programs may be substantial-initial efforts should focus on reducing viral transmission through biosecurity measures such as: (i) immediate separation of offspring from progenitors after birth followed by artificial feeding management; (ii) segregation of infected animals; (iii) periodic SRLV screening; and (iv) acquisition of animals from certified SRLV-free flocks. In low-prevalence flocks, more stringent measures, such as culling seropositive animals, may be considered to eliminate the disease. However, producer motivation remains a critical determinant of the success of any SRLV control program. Therefore, the immediate economic and productive benefits associated with disease control should be clearly emphasised to encourage adherence and long-term commitment [18,48,56,73,74].

Some limitations should be considered when interpreting these findings. The cross-sectional nature of this study identifies associations but precludes causal inference. Although a validated commercial ELISA was employed, no single diagnostic test represents a definitive gold standard for SRLV detection. Furthermore, despite the random selection of livestock farms, participation was voluntary, and, as in any field study, the potential for residual confounding cannot be entirely ruled out.

## 5. Conclusions

SRLV infection is endemic in Portugal. This study provides updated national-level epidemiological evidence, contributing to a better understanding of SRLV distribution under field conditions. The multivariable mixed-effects logistic regression model identified the following individual-level risk factors as significantly associated with increased odds of seropositivity: caprine species, non-autochthonous breed, animals older than two years, dairy aptitude, unknown serostatus of newly acquired animals, and participation in livestock competitions.

These findings offer practical guidance for developing targeted control strategies tailored to Portuguese production systems, particularly by prioritising high-risk flocks and strengthening biosecurity measures. Early laboratory-based detection, particularly serological testing, is essential for implementing targeted preventive measures. Furthermore, the results support the design and strengthening of voluntary control and eradication programs at the national level, providing evidence to guide veterinary authorities and stakeholders in decision-making. These findings support the role of veterinary authorities in promoting and monitoring voluntary control and eradication programs to effectively manage SRLV in Portuguese sheep and goat flocks.

## Figures and Tables

**Table 1:** Seroprevalence of SRLV in Portuguese flocks.

Flocks	National		Regional			
			Northern/Central		South	
	n	Positive (%)	n	Positive (%)	n	Positive (%)
Sheep	33	14 (42.42)	11	10 (90.91)	22	4 (18.18)
Goats	20	15 (75.00)	14	10 (71.43)	6	5 (83.33)
Mixed	6	4 (66.67)	3	3 (100.00)	3	1 (33.33)
Total	59	33 (55.93)	28	23 (82.14)	31	10 (32.26)

**Table 2:** SRLV seroprevalence at the individual level in Portugal.

Flocks	National		Regional			
			Northern/Central		South	
	n	Positive (%)	n	Positive (%)	n	Positive (%)
Sheep	652	124 (19.02)	241	96 (39.83)	411	28 (6.81)
Goats	380	216 (56.84)	253	135 (53.36)	127	81 (63.78)
Total	1032	340 (32.95)	494	231 (46.76)	538	109 (20.26)

**Table 3:** SRLV seroprevalence according to the animal and flock characteristics and management.

Variables	n	Positive (%)	*p*-Value
**Animal**			
Species			
Caprine	380	216 (56.84)	**<0.001**
Ovine	652	124 (19.02)	
Breed			
Non-autochthonous	583	252 (43.22)	**<0.001**
Autochthonous	449	88 (19.60)	
Sex			
Female	980	326 (33.27)	>0.05
Male	52	14 (26.92)	
Animal age			
>2 years old	722	255 (35.32)	**<0.05**
<2 years old	310	85 (27.42)	
**Location**			
Region			
Northern/Central	494	231 (46.76)	**<0.001**
South	538	109 (20.26)	
**Farm**			
Production System			
Non-extensive	607	306 (50.33)	**<0.001**
Extensive	425	34 (8.02)	
Production purpose			
Milk	575	312 (54.26)	**<0.001**
Meat	457	28 (6.13)	
Flock size			
>100 animals	745	273 (36.64)	**<0.001**
<100 animals	287	67 (13.34)	
Full confinement system			
Yes	330	190 (57.58)	**<0.001**
No	702	150 (21.37)	
Contact between sheep and goats			
Yes	361	119 (32.96)	>0.05
No	671	221 (32.94)	
**Producer**			
Farming activity type			
Hobby/Secondary	120	50 (41.67)	**<0.05**
Professional	912	290 (31.80)	
Farmer aged > 65 years old			
Yes	203	32 (15.76)	**<0.001**
No	626	268 (42.81)	
Formal training in livestock production			
No	709	188 (26.52)	**<0.001**
Yes	323	152 (47.06)	
High school education			
No	516	182 (35.27)	>0.05
Yes	516	158 (30.62)	
Producer knowledge of disease			
No	633	151 (23.85)	**<0.001**
Yes	399	189 (47.37)	
**Biosafety**			
Routine veterinary services			
No	543	150 (27.62)	**<0.001**
Yes	489	190 (38.85)	
Purchase of SRLV-negative animals			
Not know	942	321 (34.08)	**<0.05**
Yes	90	19 (21.11)	
Isolation of sick animals			
No	704	219 (31.11)	>0.05
Yes	328	121 (36.89)	
Artificial milk feeding			
No	807	179 (22.18)	**<0.001**
Yes	225	161 (71.56)	
Isolation of lambs at birth			
No	801	252 (28.60)	**<0.001**
Yes	151	88 (58.28)	
Participation in livestock competitions			
Yes	100	47 (47.00)	**<0.01**
No	932	293 (31.44)	

Bold in column “*p*-Value” is used to highlight the risk factor and in significant *p*-value.

**Table 4:** Risk factors associated with SRLV seropositivity based on mixed-effects logistic regression analysis (flock included as random effect).

Risk Factor	Univariable Mixed-Effects OR (95% CI)	Multivariable Mixed-Effects OR (95% CI)	*p*-Value
Species: goat	8.40 (2.89–24.40)	2.47 (1.01–6.03)	**<0.05**
Non-autochthonous breed	3.64 (1.13–11.74)	2.95 (1.23–7.06)	**<0.05**
Animal age > 2 years old	2.15 (1.39–3.32)	1.95 (1.29–2.94)	**<0.01**
Region: Northern/Central	6.79 (2.20–20.91)	-	>0.05
Non-extensive production system	21.20 (7.51–59.89)	2.87 (0.97–8.48)	**>0.05**
Dairy production flocks	30.53 (11.96–77.94)	8.15 (2.53–26.24)	**<0.001**
Flock size > 100 animals	1.98 (0.55–7.17)	-	>0.05
Animals in permanent confinement	9.56 (3.05–29.99)	-	>0.05
Non-professional producer	1.65 (0.29–9.42)	-	>0.05
Absence of Veterinary assistance	0.38 (0.11–1.26)	-	>0.05
Unknown serostatus of newly acquired animals	1.47 (0.23–9.36)	9.41 (2.93–30.23)	**<0.001**
Participation in livestock competitions	3.01 (0.51–17.67)	4.25 (1.42–12.73)	**<0.01**

Footnote: OR, odds ratio; CI, confidence interval. Mixed-effects logistic regression models were fitted at the individual level, with flock included as a random effect to account for intra-cluster correlation. Bold in column “*p*-Value” is used to highlight the risk factor and in significant *p*-value.

## Data Availability

The original contributions presented in this study are included in the article. Further inquiries can be directed to the corresponding author.
